# Genomic amplification of chromosome 20q13.33 is the early biomarker for the development of sporadic colorectal carcinoma

**DOI:** 10.1186/s12920-020-00776-z

**Published:** 2020-10-22

**Authors:** Vo-Minh-Hoang Bui, Clément Mettling, Jonathan Jou, H. Sunny Sun

**Affiliations:** 1 Institute of Basic Medicine, College of Medicine, National Cheng Kung University, Tainan, 701 Taiwan; 2 Department of Histology, Embryology and Pathology, University of Medicine and Pharmacy at Ho Chi Minh City, Ho Chi Minh City, Vietnam; 3 Institut de Génétique Humaine, Unité Propre de Recherche 1142 du Centre National de la Recherche Scientifique, 34396 Montpellier Cedex 5, France; 4 College of Medicine, University of Illinois, Champaign, IL 61820 USA; 5 Institute of Molecular Medicine, College of Medicine, National Cheng Kung University, 1 University Road, Tainan, 70101 Taiwan

**Keywords:** Chromosome instability, Microsatellite instability, Biomarker, Sporadic CRC, Adenoma – carcinoma process

## Abstract

**Background:**

Colorectal carcinoma (CRC) is the third most common cancer in the world and also the third leading cause of cancer-related mortality in Taiwan. CRC tumorigenesis is a multistep process, starting from mutations causing loss of function of tumor suppressor genes, canonically demonstrated in adenomatous polyposis coli pathogenesis. Although many genes or chromosomal alterations have been shown to be involved in this process, there are still unrecognized molecular events within CRC tumorigenesis. Elucidating these mechanisms may help improve the management and treatment.

**Methods:**

In this study, we aimed to identify copy number alteration of the smallest chromosomal regions that is significantly associated with sporadic CRC tumorigenesis using high-resolution array-based Comparative Genomic Hybridization (aCGH) and quantitative Polymerase chain reaction (qPCR). In addition, microsatellite instability assay and sequencing-based mutation assay were performed to illustrate the initiation event of CRC tumorigenesis.

**Results:**

A total of 571 CRC patients were recruited and 377 paired CRC tissues from sporadic CRC cases were used to define the smallest regions with chromosome copy number changes. In addition, 198 colorectal polyps from 160 patients were also used to study the role of 20q13.33 gain in CRC tumorigenesis. We found that gain in 20q13.33 is the main chromosomal abnormalities in this patient population and counts 50.9 and 62.8% in CRC and colon polyps, respectively. Furthermore, *APC* and *KRAS* gene mutations were profiled simultaneously and co-analyzed with microsatellite instability and 20q13.33 gain in CRC patients. Our study showed that the frequency of 20q13.33 copy number gain was highest among all reported CRC mutations.

**Conclusion:**

As *APC* or *KRAS* mutations are currently identified as the most important targets for CRC therapy, this study proposes that 20q13.33 copy number gain and the associated chromosomal genes function as promising biomarkers for both early stage detection and targeted therapy of sporadic CRCs in the future.

## Background

Colorectal carcinoma (CRC) is the third most common cancer affecting over 1 million people and the fourth leading cause of cancer-related mortalities worldwide [1]. The American Cancer Society estimates that 71,420 men and 64,010 women were diagnosed and 27,150 men and 23,110 women died of CRC in 2017 in the US (Date accessed: 20170304, [2]). In Taiwan, CRC is the third leading cause of cancer mortality after lung and liver cancers in 2018 (Date accessed: 20190720, [3]).

Two types of genomic mutational events are associated with CRC tumorigenesis: chromosomal and microsatellite instabilities (CIN and MSI). Over 90% of CRCs are sporadic [4], with CIN representing the most common type of genomic instability in sporadic CRC [5]. CIN is characterized by aneuploidy, chromosomal rearrangements, and accumulated somatic mutations in *KRAS* oncogenes and *APC* and *p53* tumor suppressor genes [6]. Conversely, the molecular mechanism of MSI results from the loss of function of the DNA mismatch repair (MMR) system [5]. While the molecular pathogenesis of MSI is well documented, the specific molecular events resulting in CIN is less clear and theorized as chromosomal alterations leading to the loss of tumor suppressor genes and the gain of oncogenes.

To illustrate the molecular events associated with CIN, previous studies used metaphase CGH or bacterial artificial chromosome (BAC)-based aCGH to identify DNA copy number alterations in CRC [7–12]. However, because the detection limit of metaphase CGH is around 10–20 Mb, the resolution is too low to detect micro-genomic alterations below 30 kb [13]. Promising higher resolution, BAC-based aCGH techniques were applied to screen DNA copy numbers. Nevertheless, its large size limited resolution and need of BAC clones increased time and associated costs [13].

Oligo-based aCGH has been introduced with the advantages of flexibility in probe design, greater coverage, and higher resolution [13]. Wicker et al. [13] showed that the oligo-based aCGH detected 209 amplifications and 314 deletions among the 19 advanced prostate cancer samples whereas the BAC platform only detected 74 amplifications and 71 deletions. Furthermore, the oligo-based aCGH has been used for high-throughput detection of DNA copy number alterations in the entire human genome [14, 15]. Besides identifying the chromosomal gains and losses previously detected using mCGH or BAC-based aCGH, the high-resolution oligo-based aCGH can detect minimum common regions as well, which provides an effective way to narrow candidate genes [15]. For example, an oligo-based aCGH analysis uncovered a 0.34-Mb submicroscopic deletion at chromosome 5q31.1 within a complementary determining-region in a normal karyotype AML patient which demonstrated prognostic significance [16]. In this study, we applied an ultra-dense (1 million probe) oligomer-based aCGH technology to identify minimal common regions of DNA copy number alteration associated with sporadic CRC candidate genes. Our data revealed that chromosome 20q13.33 gain is the earliest mutational event found in the majority of CRC and colon polys, thus it suggests Chromosome 20q13.33 amplification as an early biomarker for adenoma – carcinoma process in the development of sporadic CRC.

## Results

### Frequencies of microsatellite instabilities in sporadic CRC

We applied a modified pentaplex PCR procedure to assay the status of microsatellite instability in our sporadic CRC samples. Among 571 CRC cases, 377 are sporadic CRCs and used for further investigation. We identified 38 microsatellite instable high (MSI-H,10.1%), 58 microsatellite instable low (MSI-L, 15.4%), and 281 microsatellite stable (MSS, 74.5%) CRCs. Combining clinicopathological features with MSI status, significant correlations between MSI status and gender (*P* <  0.05), age (*P* <  0.02), tumor location (*P* < 0.001), and tumor differentiation (*P* < 0.05) were found (Table 1). Nevertheless, no correlation was detected between the AJCC stage and these three groups of microsatellites. These data showed that MSI-H CRCs were more likely to occur at the right sided (63.2%) of the colon, while MSS CRCs were more frequent found at the left side (71.2%) of the colon.

**Table 1:** Clinicopathological features of sporadic CRC patients included in this study

Variables	Number (%)	MSI-H	MSI-L	MSS	***P*** value
No. of patients	377	38	58	281	
**Gender**					**0.042**
Male	219 (58.1)	23	25	**171**	
Female	158 (41.9)	15	**33**	110	
**Age**					**0.007**
< 50	49 (13)	11	5	33	
≥ 50	328 (87)	27	53	248	
**AJCC stage**					0.925
Early (stage I/II)	173 (45.9)	19	25	129	
Late (stage III/IV)	202 (53.6)	21	32	149	
Both	2 (0.5)	1	1	0	
**Location of tumor**					**< 0.001**
Right	124 (32.9)	**24**	17	83	
Left (including rectum)	253 (67.1)	14	41	**198**	
**Differentiation of tumor**					**0.014**
Poor	38 (10.1)	**9**	8	21	
Moderate	294 (78)	27	41	**226**	
Well	45 (11.9)	2	9	34	

### Copy number alterations in sporadic CRC samples detected by aCGH

**Fig. 1:**
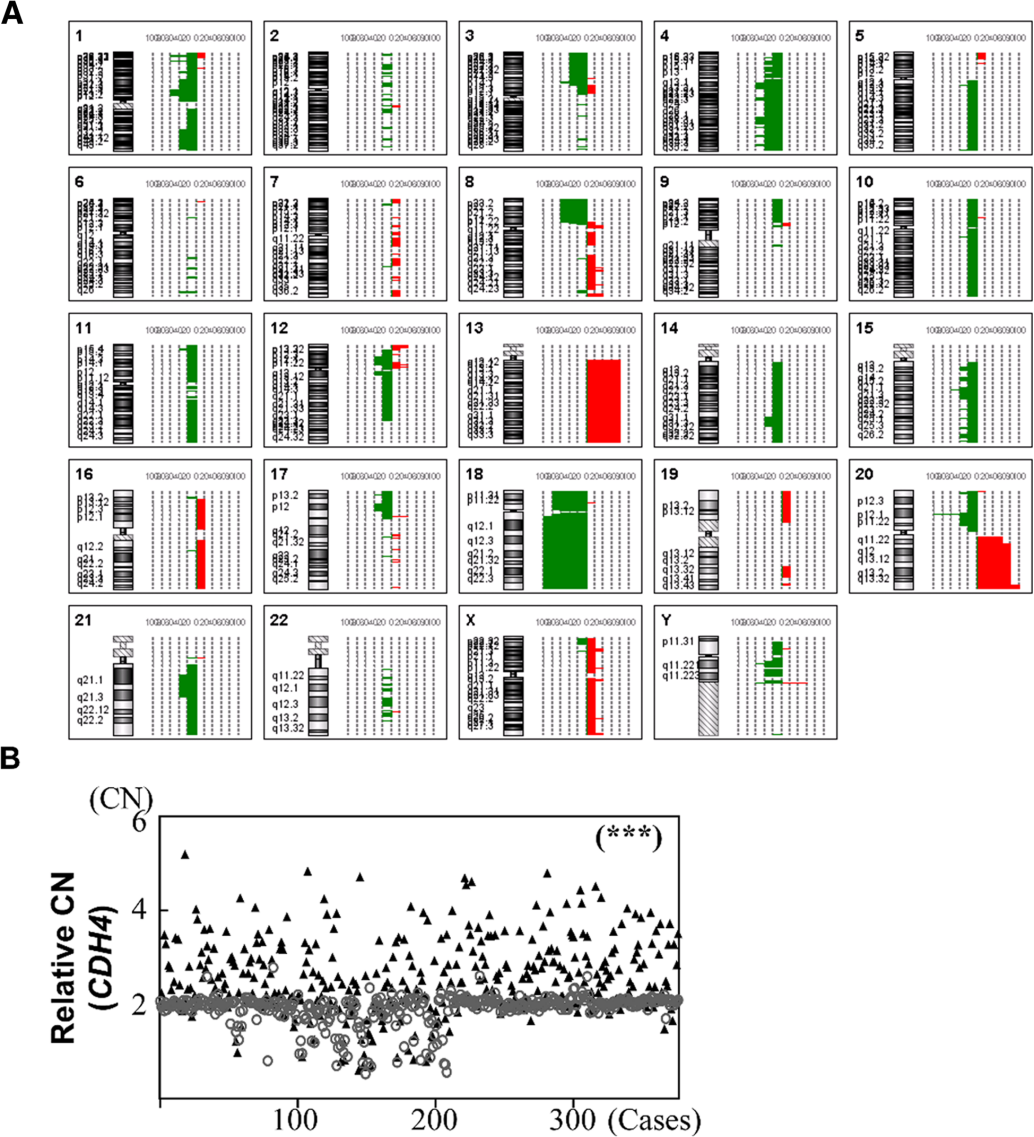
Profiles of chromosomal aberrations in sporadic CRC. **a** Overall frequency of chromosomal copy number aberrations detected by a high resolution aCGH (Agilent 1x1M oligonucleotide probes) in sporadic CRCs (*n* = 5). All gain or loss events were identified using log_2_ ratios of fluorescent signals between labeled tumor and adjacent non-tumor parts of CRCs. **b** Scatter plots were used to show quantitative real-time PCR analyses of *CDH4* gene loci for 377 sporadic paired CRC tissues. The CRC tumor parts were denoted by black triangles, whereas the non-tumor ones were denoted by unfilled circles. Both parts were compared to detect whether copy number alterations of CRC tumors could be correlated with the genomic alterations of the adjacent CRC non-tumors (correlation coefficient *R*^2^ < 0.01). * *P* < 0.05, ** *P* < 0.01, and *** *P* < 0.001

Genomic DNAs from 5 sporadic CRC cases were selected to detect copy number variation using high-resolution aCGH. We detected a total of 204 chromosomal variations in these 5 sporadic CRC cases (Fig. 1a and Additional File 1: Table S1). The sizes of altered regions vary significantly, ranging from 3 probes to ~ 18,000 probes. Interestingly, MSS cases tended to have more chromosomal gains than losses (average ratio 1.8 to 1). On the other hand, microsatellite unstable CRC showed an opposite phenomenon with fewer chromosomal gains and more chromosomal deletions (*P* < 0.05).

At a region-specific level, we found the entire chromosome 4 and whole arm of chromosomes 13q, 14q, 18q and Xq were altered in our sporadic CRC cases. Among them, 9 regions on five chromosomes were found to be altered in at least 4 cases (Table 2) and chromosome 20q13.33 gain (4.5 Mb) was the smallest chromosome alteration found in all 5 cases thus was selected for further examination.

**Table 2:** Common copy number alterations in 5 sporadic CRCs

Number	Chromosome	Cytoband	Start position	Stop position	Size (bp)	Number of observations	Number of probes	Variant type
1	18	q11.2-q23	17,303,810	76,116,083	58,812,273	5	87,610	Loss
2	20	p12.1	11,902,811	17,799,351	5,896,540	5	4085	Loss
3	20	q13.33	57,900,503	62,419,593	4,519,090	5	8692	Gain
4	1	p36.23	7,100,548	9,199,731	2,099,183	4	2191	Gain/Loss
5	4	q22.1	88,201,557	93,996,406	5,794,849	4	4593	Loss
6	13	q12.11-q34	18,402,053	114,118,329	95,716,276	4	127,133	Gain
7	18	p11.21-p11.32	4316	15,069,335	15,065,019	4	11,757	Loss
8	20	p13	12,719	4,998,099	4,985,380	4	2103	Gain/Loss
9	20	q11.23-q13.32	33,900,333	57,898,212	23,997,879	4	34,429	Gain

### Quantitative measurement of frequency of chromosome alternations in CRCs

**Fig. 2:**
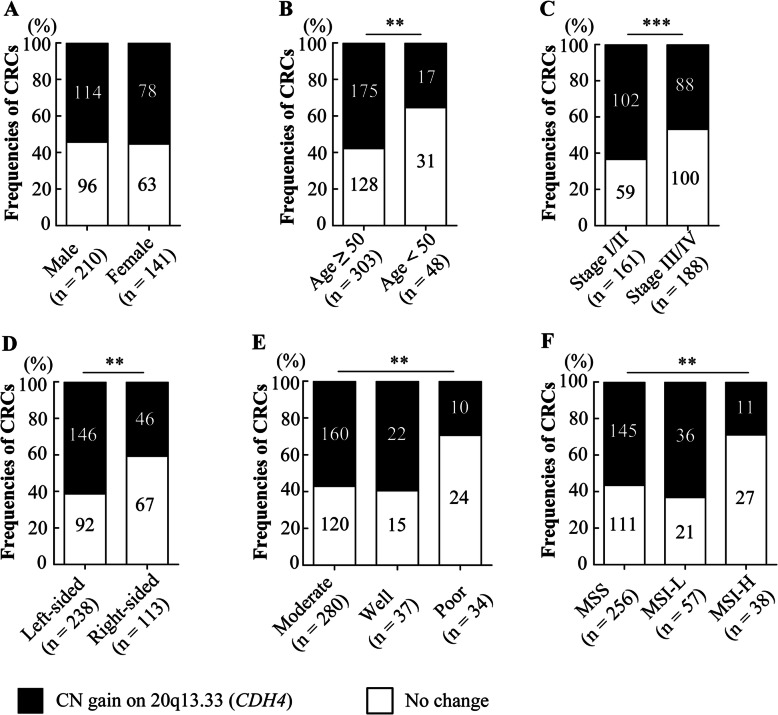
Association of 20q13.33 gain and clinicopathological features in CRC patients. Clinicopathological features are gender (**a**), age (**b**), AJCC stage including early (I / II) and late (III / IV) stages (**c**), location (**d**) as well as differentiation level of tumor (**e**), and status of microsatellite instability (**f**). ** *P* < 0.01, *** *P* < 0.001

To study the significance of chromosome 20q13.33 gain in CRC development, we designed TaqMan copy number quantitative PCR (qPCR) assays for the Cadherin-4 (*CDH4*) that is located in the middle of the 20q13.33 target region to detect chromosomal gain (Additional File 2: Fig. S1). qPCR assays were performed in 377 paired CRC samples. Results from the *CDH4* copy number qPCR assay showed gains of *CDH4* copy numbers in 50.9% of CRC tumors compared with only 1.3% of the adjacent normal tissue (Additional File 3: Table S2, Fig. 1b, *P* < 0.001). To determine the clinical significance of chromosome 20q13.33 amplifications, we compared *CDH4* copy number gain samples with various clinicopathological features in 377 CRC cases (Fig. 2). We detected significant differences in the presence of *CDH4* copy number gain between age groups (*P* < 0.01), tumor locations (*P* < 0.01), AJCC stages (*P* < 0.001), differentiation level (*P* < 0.01) and microsatellite stability status (*P* < 0.01).

The consequences of chromosome 20q13.33 amplification on CRC tumorigenesis were investigated. Although the copy number of 20q13.33 region was unknown, we analyzed gene expression in CRC samples using TCGA database. While expressions of CDH4 are not changed between tumor and normal tissues (Additional File 2: Fig. S2A, left), CDH4 expression is found to be significantly associated with overall survival (*P* < 0.01; Additional File 2: Fig. S2B, left). On the other hand, one putative oncogene located very close to CDH4 on 20q13.33 region, ADRM1, was found to be significant overexpressed in the CRC tissues (*P* < 0.05; Additional File 2: Fig. S2A, right) but not be associated with patients’ survival (Additional File 2: Fig. S2B, right). These data imply, at least in part, genes located within 20q13.33 region contribute individually or jointly to various aspects of CRC tumorigenesis.

### 20q13.33 copy number gain is not a germline mutation

Although the genomic alterations of CRC non-tumor tissues were distinct from those of their corresponding tumors, we noticed that 42 CRCs (11.1%) with *CDH4* copy number loss in non-tumor tissue. Whether the copy number loss on chromosome 20q13.33 represents germline mutations were investigated. Twenty-six available blood samples (Additional File 4: Table S3) from 42 sporadic CRC cases demonstrating *CDH4* copy number loss in non-tumor tissue were collected and assayed with *CDH4* copy number qPCR. Interestingly, no *CDH4* copy number change was found in these blood samples (Additional File 3: Table S2). Moreover, we performed *CDH4* copy number qPCR on DNAs from 94 population samples of Han Chinese and found no gain or loss in this chromosomal region. Taken together, these data clearly demonstrate that chromosome 20q13.33 region is not a common copy number variation (CNV) site for chromosomal alteration. Nevertheless, the fact that copy number alteration on chromosome 20q13.33 is restricted to the somatic cells merits further investigation to elucidate the biological significance of these amplifications in CRC tumorigenesis.

### 20q13.33 copy number gain in colon polyps

To study the biological significance of 20q13.33 gain in the initiation of CRC tumorigenesis, we investigated this somatic event in 198 colon polyps from 160 individuals who underwent standard colonoscopy examination at the National Cheng Kung University Hospital. The classifications of these colon polyps are given in Table 3. Overall, 133 (67.2%) samples were adenomatous polyps, with tubular adenoma as the most common type (> 60%). There were 33 (16.7%) serrated polyps including hyperplastic polyps (17) and serrated adenomas (16) collected. In addition, we also found 32 (16.1%) confirmed adenocarcinomas on pathology examination.

**Table 3:** Pathological diagnoses of colon polyps included in this study

Pathological diagnosis	Number (%)	Single polyp	Multiple polyp
		Number (%)	Number (%)
Adenomatous polyps	133 (67.2)	96 (73.3)	37 (55.2)
+ Tubular adenoma	80 (40.4)	59 (45.0)	21 (31.3)
+ Tubulovillous adenoma	40 (20.2)	27 (20.6)	13 (19.4)
+ Villous adenoma	13 (6.6)	10 (7.7)	3 (4.5)
Serrated polyps	33 (16.7)	18 (13.7)	15 (22.4)
+ Hyperplastic polyp	17 (8.6)	8 (6.1)	9 (13.4)
+ Serrated adenoma	16 (8.1)	10 (7.6)	6 (9.0)
Adenocarcinoma	32 (16.1)	17 (13.0)	15 (22.4)
**Total**	**198 (100)**	**131 (100)**	**67 (100)**

As summarized in Table 4, qPCR of *CDH4* copy number revealed that over 60% (124 / 198) of colon polyps demonstrated 20q13.33 amplification. The phenomenon is even more profound in the serrated polyps, where the highest frequency of 20q13.33 gain occurs in hyperplastic polyps 82.4% (14/17), and followed by Serrated adenoma with 68.8% (11/16) frequency. On the other hand, tubulovillous adenomas (19/40 = 47.5%) has the lowest rate of 20q13.33 gain. The high frequency of colon polyps in association with 20q13.33 gain suggested that this chromosomal alteration is a mutational event occurring in early CRC tumorigenesis and may participate in the initiation of CRC development.

**Table 4:** Features of genomic alterations in colon polyps

Genomic alterations	Pathological diagnoses of colon polyps / (N)					
	AC/	HYP/	SA/	TA/	TVA/	VA/
	32	17	16	80	40	13
***APC*** **mutation**						
+ Frameshift	10	1	0	19	13	5
+ Nonsense	5	1	2	15	8	1
+ Nonsense and Frameshift	1	0	0	0	0	0
+ Missense and Frameshift	0	0	0	1	1	0
+ Nonsense and Missense	0	0	0	2	0	0
**Total**	**16**	**2**	**2**	**37**	**22**	**6**
***KRAS*** **mutation**						
+ Codon 12	8	2	2	4	13	6
+ Codon 13	2	0	0	3	2	4
+ Both	1	0	0	0	0	0
**Total**	**11**	**2**	**2**	**7**	**15**	**10**
**20q13.33 copy number**						
+ Loss	0	0	0	3	1	0
+ Gain	20	14	11	52	19	8
**Total**	**20**	**14**	**11**	**55**	**20**	**8**
**MSI status**						
+ MSS	22	12	10	67	29	8
+ MSI-L	4	1	2	12	6	4
+ MSI-H	6	4	4	1	5	1
**Total**	**32**	**17**	**16**	**80**	**40**	**13**

*Abbreviations*: *AC* Adenocarcinoma, *HYP* Hyperplastic polyp, *SA* Serrated adenoma, *TA* Tubular adenoma, *TVA* Tubulovillous adenoma, *VA* Villous adenoma

### Mutation assays of MSI, KRAS and APC in colon polyps

It is known that MSI, *KRAS* and *APC* mutations are the early mutational events in CRC tumorigenesis. To study the impact of these well-known mutations in our CRC cases, we performed MSI assay together with mutation analysis of *APC* and *KRAS* in 198 colon polyps. As listed in Table 4, a majority (148/198 = 74.7%) of the colon polyps was classified as MSS and the overall distribution among 3 classes in colon polyps was similar to the pattern observed in 377 CRCs.

*APC* exon 15 and *KRAS* exon 2 mutation analyses were performed in all colon polyps which demonstrated that nearly a half of samples exhibited *APC* exon 15 mutations (42.9%, 85 polyps). Among these, frameshift mutations predominated (48 polyps, 56.5%), followed by nonsense mutations (32 polyps, 37.6%) and mixed type (5 polyps, 5.9%). In contrast, *KRAS* exon 2 mutations were observed in less than 25% polyps (23.7%, 47 polyps) with the most common mutation at codon 12 (74.5%).

### Mutational profile of chromosome 20q13.33 gain, MSI, KRAS and APC in colon polyps

**Fig. 3:**
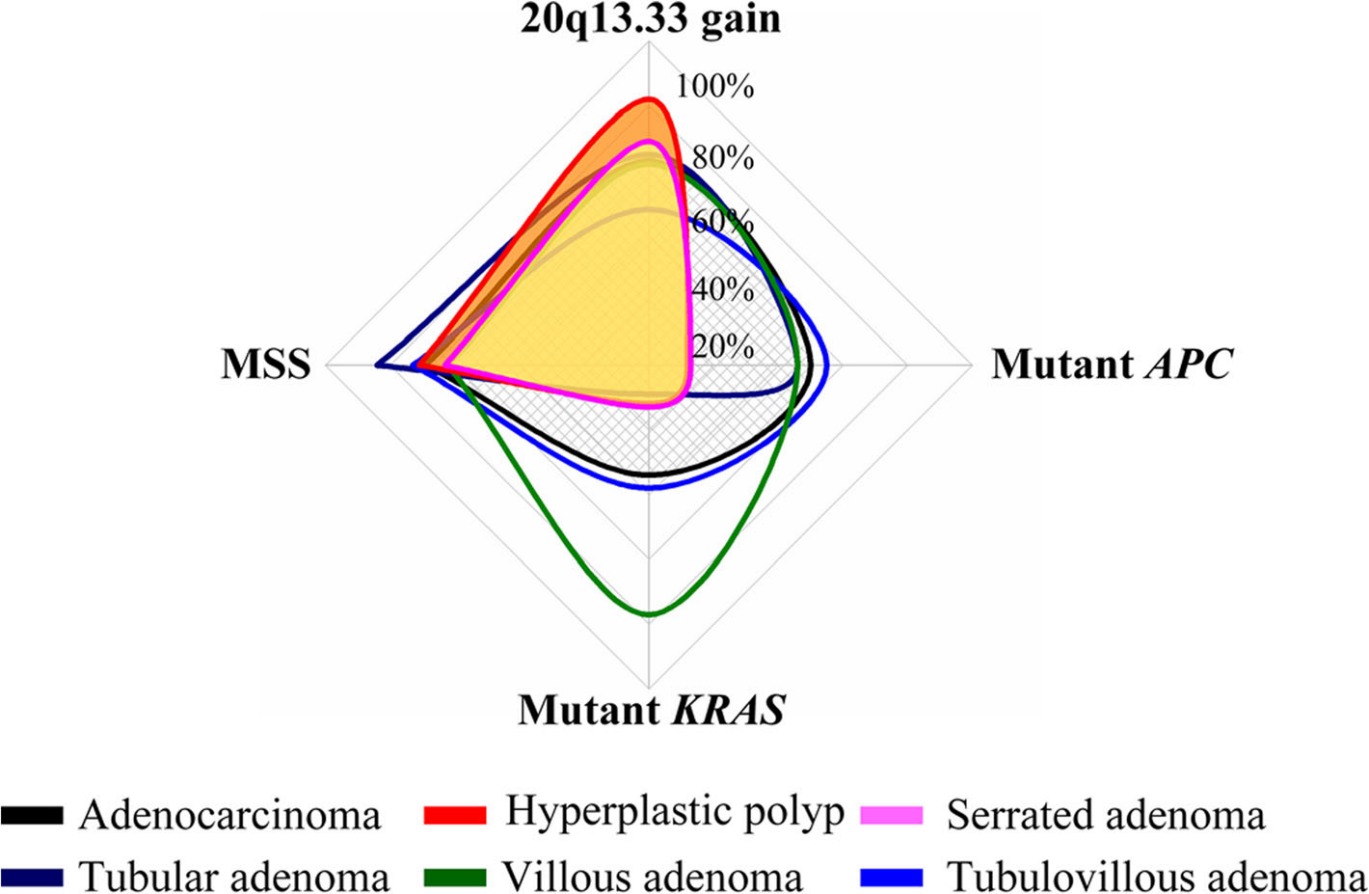
Profiles of genomic alteration events in colon polyps. (*n* = 198). The profile was established using mutational profiles from 198 colon polyps. The main genomic alterations of *APC* mutation, *KRAS* mutation, MSI and 20q13.33 gain were presented

To clarify the role of chromosome 20q13.33 gain as initial event in different lineages of colorectal tumorigenesis, especially in relation to other somatic events like *APC* or *KRAS* mutations, we analyzed all mutational events obtained in 198 polyps together and applied a multi-measure radar chart to demonstrate the mutational profiles of different polyp types (Fig. 3). Our data showed two distinct mutational profiles of adenomatous and serrated polyps. In agreement with current literature, our mutation profile demonstrated that hyperplastic polyps and serrated adenomas share similar mutational features, characterized by a high frequency of 20q13.33 copy number gain and low frequencies of other types of mutations (i.e., MSI, APC and KRAS mutations). On the other hand, adenomatous polyps presented a dissimilar profile that shows high frequencies of mutant *APC* and MSI together with chromosome 20q13.33 gain. While the overall mutational profiles are similar, villous adenomas presented the highest level of *KRAS* mutations (76.9%). Interestingly, we observed the mutation profile of tubulovillous adenomas was nearly the same as that of adenocarcinomas. Comparing the frequency of 20q13.33 copy number gain with other mutations, except villous adenomas, most polyps presented a higher frequency of 20q13.33 copy number gain than other mutations. Overall, our data indicates that copy number gain on chromosome 20q13.33 is the most common early mutational event in the adenoma-carcinoma sequence and suggests that it may represent an initial biomarker for CRC tumorigenesis.

## Discussion

MSI, especially MSI-H, was identified as a clinical practice requirement in the understanding of CRC pathogenesis and prevention [17]. Depending upon applied methods to detect MSI, approximately 11–17% of CRCs demonstrate MSI (or MSI-H) [18, 19], with only 3% Lynch syndrome concurrent [20]. Nevertheless, most detected MSI CRCs are sporadic [20].

To uncovering the structural chromosomal instabilities in CRC tumorigenesis, previous studies had used metaphase CGH (mCGH) or bacterial artificial chromosome (BAC)-based aCGH to identify DNA copy number alterations in CRC [12, 21]. Due to the detection limit of mCGH is around 10–20 Mb, it was considered to be too low resolution to detect micro-genomic alterations (< 30 kb) [13] . In this study, we applied an oligonucleotide-based aCGH microarray chip to investigate chromosomal alteration in sporadic Taiwanese CRCs. The rate of MSI-H and non-MSI-H CRCs in our sporadic CRCs was 10.9 and 89.1%, respectively, which is similar to those of previous studies [18, 22]. In addition, the MSI status in association with various clinicopathological features, for example, in younger patients [23] and right-sided and poorly differentiated tumors [18], are similar as previous reports. By using high-resolution aCGH to analyze 5 representative CRC cases, we showed the most common chromosomal alterations were gains of 13q12.11-13q14 (95.7 Mb), 20q11.23-20q13.32 (24 Mb), 20q13.33 (4.5 Mb) and losses of 18p11.21-18p11.32 (12.7 Mb), 18q11.2-18q23 (58.8 Mb), 4q22.1 (5.8 Mb), 20p12.1 (5.9 Mb). Among them, the losses detected in 4q22.1 or 20p12.1 have not been reported previously using different platforms [24, 25].

All 5 representative CRC cases (including 1 MSI-H, 1 MSI-L and 3 MSS) exhibited copy number alterations. In contrast to MSS CRCs, we found more copy number losses than gains in MSI CRCs. This finding contradicted previous studies, which claimed MSI CRCs were diploid or low copy number alterations [26, 27]. The difference may be due to the use of a high-resolution oligonucleotide-based aCGH in this study thus it enable us to identified small genomic alterations that have not being detected previously. Moreover, we also found copy number gains on chromosome 8q24.3, Xp11.23, Xq13.1, Xq25 and Xq28 and losses on chromosome Yq11.222-Yq11.223 were frequently found in MSS but not in MSI CRCs. Interestingly, copy number losses are predominately found in two chromosomes: chromosomes 1 (1p13.3-1p22.1 and 1p36.12) and 4 (4q12-4q13.2, 4q21.3, 4q28.1-4q28.3, 4q31.22, 4q32.2 and 4q34.6) in MSI but not in MSS CRCs. An early study has reported copy number losses in MSI CRCs on chromosome 4q [28], our high-resolution aCGH indeed provides more detailed regional changes on human chromosome 4 in CRC. Nevertheless, the biological significance of chromosome 4 deletion on CRC tumorigenesis merits further investigation.

We performed TaqMan Copy Number assay using qPCR to validate chromosomal alterations and continued to investigate the biological significance of 20q13.33 copy number gain. We found 20q13.33 gain was closely associated with clinicopathological features, including old patients (age over 50), MSS, AJCC stage I, left-sided, and moderate colon tumors (*P* < 0.01). These results suggest that gain of 20q13.33 may be specific for elderly CRC patients and contribute to the localization and differentiation of CRC tumors as well as tumorigenesis. As the genomic alteration was not found in the DNAs from 26 CRC patients’ blood whose CRC showed 20q13.33 gain, and 94 Han Chinese population samples, these results indicate that 20q13.33 copy number alteration is a somatic event.

To clarify the role of 20q13.33 copy number gain as an initial event in CRC tumorigenesis, especially in relation to other early somatic events, such as *APC* or *KRAS* mutations, we analyzed all mutational events as well as the MSI status of all 198 polyps combined. Using the *CDH4* gene as a representative marker of 20q13.33, we found that 20q13.33 copy number gain is even more prevalent in colon polyps than in the CRCs (62.6% vs 50.9%, respectively). While most of our serrated polyps were *KRAS* mutation negative and microsatellite stable, we found that the frequency of 20q13.33 gain in serrated polyps was 1.3-fold higher compared to the adenomatous polyps (25/33 vs 79/133, respectively). In addition, the mutation rate of 20q13.33 copy number gain was higher than *APC* mutation (62.63% vs 42.93%, respectively). The data suggest that 20q13.33 copy number gain is an early genomic event in the pathway of CRC tumorigenesis. Furthermore, our data identified two distinct mutation profiles which correlated to adenomatous and serrated polyps. While the mutation profile of serrated polyps demonstrated high frequencies of 20q13.33 copy number gain and MSI, this type of polys lacks both *APC* and *KRAS* mutations. On the other hand, adenomatous polyps displayed high frequencies of all mutational events including 20q13.33 gain. Among them, Villous adenomas presented higher frequencies of *KRAS* mutations and MSI compared to the tubular adenomas. Therefore, this characteristic may contribute to the more malignant nature of villous adenomas compared to adenomatous polyps (Shinya and Wolff 1979). Nevertheless, our study did not distinguish pathological classifications of serrated polyps, such as sessile serrated or traditional serrated polyps. Therefore, we cannot conclude which subtypes of serrated polyps present a higher frequency of 20q13.33 gain. Further studies with detailed colon polyp classification criteria on a large sample may confirm our findings.

Gains of 20q are commonly observed in various type of cancers including CRCs [29] and breast cancer [30]. In addition, copy number gain of 20q13 was shown to be associated with the adenoma-carcinoma process [31]. Using aCGH combining with microarray analysis, previous study has identified multiple putative oncogenes on chromosome 20q that are important in chromosomal instability-related adenoma to carcinoma progression [32]. Among these putative oncogenes, ADRM1, C20orf20 and TCFL5 are located in chromosome 20q13.33 region. As shown in Figure S1, chromosome 20q13.33 region, especially from 60 Mb to 63 Mb interval are gene-rich region and all three putative oncogenes are clustered together with CDH4. It is possible the initial genomic alteration of chromosome 20q13.33 region affects the expression of few or even all of these putative oncogenes. The changed expression of them thus drives the cells toward tumorigenic initiation and progression. Our study provides a biomarker to detect early stage genomic alteration, thus provide a value for early dection. Nevertheless, the causes that lead to copy number gain of chromosome 20q13.33 and the biological significance of this alteration remains to be elucidated in details in the future.

## Conclusion

Using 377 paired CRC tissues from 571 patients and 198 colorectal polyps from 160 patients, this study discovered chromosome 20q13.33 amplification is the most significant copy number alteration in association with early sporadic CRC tumorigenesis. By comparing the genomic alteration of 20q13.33 gain with APC and KRAS mutations (62.63% vs 42.93 and 23.73%, respectively), which are currently identified as the most important targets for CRC therapy [33], the results support that 20q13.33 gain may have been involved in the development of cancer. Therefore, 20q13.33 copy number gain and the associated chromosomal genes can serve as promising biomarkers for both early stage detection and targeted therapy of sporadic CRCs in the future.

## Methods

### Sample collections

A total of 571 CRC patients who underwent surgery at National Cheng Kung University Hospital from 2005 to 2012 were recruited in this study. The stage of each tumor was classified and histologically confirmed by pathologists. This study was approved by the Clinical Research Ethics Committee at the National Cheng Kung University Medical Center, and informed consent was obtained from each patient. Cases with known familial CRC history or lack of clinicopathological information were excluded from the study. Paired normal and tumor specimens from 377 sporadic CRC patients fitting the Revised Bethesda criteria were further analyzed [34]. The clinicopathological characteristics of these CRC patients are provided in Table 1. In addition, 198 colon polyps from 160 individuals removed during colonoscopy at the National Cheng Kung University Hospital were also included in this study. A summary of polyp pathology is presented in Table 3.

To validate whether the observed genomic alterations were restricted to the somatic cells, blood samples from 26 cases demonstrating chromosome copy number alterations in normal tissue were collected from the NCKU tissue bank. Genomic DNA from 94 unrelated individuals in the Han Chinese population were obtained from the Taiwanese Han Chinese Cell and Genome Bank [35] and used as population controls.

### Microsatellite instability analysis

DNA was isolated from tumor and adjacent normal tissue in selected sporadic CRC samples using the DNA isolation kit from QIAGEN according to the manufacturer’s recommendation (QIAGEN Inc., Darmstadt, Germany). We established a procedure originally developed by Suraweera [36] and modified by Ebinger [37] to examine microsatellite status. Primers for five reference microsatellite markers include BAT-26 (hMSH2), BAT-25 (c-kit), NR-21 (SLC7A8), NR-22 (transmembrane precursor protein B5), and NR-24 (Zinc finger 2) were synthesized (IDT, Coralville, IA) and used in the pentaplex polymerase chain reaction (Additional File 5: Table S4) [37]. PCR products were size separated by capillary electrophoresis (CE) using an ABI 310 Genetic Analyzer (Applied Biosystems, Foster City, CA) and analyzed using GeneMapper (version 3.7, Applied Biosystems, Foster City, CA). Tumor samples exhibiting allele peaks different from the matched non-tumors were classified as microsatellite instability (MSI) for the given marker. High-frequency MSI (MSI-H) and low-frequency MSI (MSI-L) were called when the number of unstable markers reached 1 and ≥ 2, respectively. The cases without evidence of unstable markers were labeled as microsatellite stable (MSS). Ambiguous results were re-analyzed.

### Array comparative genomic hybridization (aCGH)

To illustrate chromosomal abnormalities at high-resolution, 50 ng of genomic DNA from tumor and adjacent normal tissue of 5 representative CRC cases (Additional File 5: Table S4) were assayed using SurePrint G3 Human CGH 1x1M Microarray (Agilent Technologies, Santa Clara, CA) according the manufacturer’s protocol. Experimental and reference DNA was labeled with Cy3-dUTP and Cy5-dUTP, respectively, followed by cleaning of the labeled targets, pooling, and mixing to perform hybridization on SurePrint G3 Human CGH 1x1M microarrays at 65 °C for 40 h. The array was washed and scanned on an Agilent DNA microarray scanner at 535 nm for Cy3 and 625 nm for Cy5 at a resolution of 3 μm. The scanned images were normalized to quantify signal and background intensity for each feature using DNA analytics 4.0 (Agilent Technologies, Santa Clara, CA).

### Quantitative real-time polymerase chain reaction (qPCR)

The TaqMan® Copy Number Assays (Applied Biosystems, Foster City, CA) was designed and used to confirm copy number variation from the aCGH results. Each reaction was triplicated in 96 well plates and run on a sequence detector (ABI StepOne Plus™; Applied Biosystems). DNA copy number was determined by using a relative quantification method (i.e., the 2^-△△Ct^ method) and normalized to the reference genes, RNaseP and TERT (TaqMan Copy Number Reference Assays). The predicted copy number (PCN) of each sample was compared to the reference samples in which the copy number were known by aCGH to determine the final DNA copy.

### *APC* and *KRAS* gene mutation analysis

To assay the mutation profile of *APC* and *KRAS* genes in CRC polyps, we performed PCR to amplify exon 2 (included codons 12 and 13) of the *KRAS* gene and the mutation cluster region located on exon 15 of the *APC* gene. The 288-base-pair (bp) PCR product of *KRAS* and 3 overlapping PCR products of 295, 533, and 300 bp from *APC* were examined by direct sequencing using the Applied Biosystems 3130 Genetic Analyzer (Applied Biosystems, Foster City, California, USA). All primers used to amplify the target regions were provided in the Additional File 5: Table S4.

### Gene expression and overall survival analysis using TCGA datasets

To study expression of selected target genes in cancer and patients’ survival, data from colon cancer (*n* = 275) and normal colon (*n* = 349) tissues were analyzed from TCGA database using GEPIA tool (http://gepia.cancer-pku.cn/index.html).

### Statistics

All test statistics were performed using Pearson Chi-Square tests implemented in the SPSS 17.0 software package (SPSS Inc., Chicago, Illinois). Fisher’s exact test was used when the expected value for one cell was lower than five. All variables were presented in frequencies and the significance level for all statistical tests was set at 0.05.

## Supplementary information

**Additional file 1: Table S1.** Clinicopathological characteristics of 5 representative CRCs.

**Additional file 2: Figures S1.** Genome coordinates and annotated genes on chromosome 20q13.33 region; and **S2.** Expression and survival analysis of CDH4 and ADRM1 in TCGA colon cancer dataset.

**Additional file 3: Table S2.** Frequencies of copy number alterations in 4 target regions.

**Additional file 4: Table S3.** Detailed clinical information and 20q13.33 copy number estimation of CRC sample showing 20q13.33 copy number loss in their non-tumor part.

**Additional file 5: Table S4.** Primers used in this study.

## Data Availability

All data presented in this study are provided either in the manuscript or additional files.

## Abbreviations

CRC: Colorectal cancer
aCGH: Array comparative genomic hybridization
qPCR: Quantitative polymerase chain reaction
CN: Copy number
CNV: Copy number variation
CNA: Copy number alteration
CIN: Chromosomal instability
MS: Microsatellite
MSI: Microsatellite instability
MSS: Microsatellite stable
MSI-L: Microsatellite instability – low
MSI-H: Microsatellite instability – high
