# Supplementary material for: Genomic amplification of chromosome 20q13.33 is the early biomarker for the development of sporadic colorectal carcinoma
Source: BMC Med Genomics. 2020 Oct 22;13(Suppl 10):149. doi: 10.1186/s12920-020-00776-z (PMC7579792; doi:10.1186/s12920-020-00776-z)
Supplement: Supplementary file 1 — Additional file 1: Table S1. Clinicopathological characteristics of 5 representative CRCs. [file 12920_2020_776_MOESM1_ESM.docx]

**Table S1.** Clinicopathological characteristics of 5 representative CRCs

| **Case** | **Clinicopathological characteristics** | | | | | |
| --- | --- | --- | --- | --- | --- | --- |
|  | **Gender** | **Age** | **Duke’s stage** | **Tumor location** | **Tumor differentiation** | **MSI status** |
| **34** | Male | 53 | C2 | Left | Moderate | MSS |
| **48** | Female | 71 | B2 | Left | Moderate | MSI-L |
| **59** | Male | 58 | B1 | Left | Moderate | MSS |
| **60** | Female | 86 | D | Right | Moderate | MSS |
| **65** | Female | 75 | C3 | Left | Moderate | MSI-H |
