# Supplementary material for: Genomic amplification of chromosome 20q13.33 is the early biomarker for the development of sporadic colorectal carcinoma
Source: BMC Med Genomics. 2020 Oct 22;13(Suppl 10):149. doi: 10.1186/s12920-020-00776-z (PMC7579792; doi:10.1186/s12920-020-00776-z)
Supplement: Supplementary file 2 — Additional file 2: Figures S1. Genome coordinates and annotated genes on chromosome 20q13.33 region; and S2. Expression and survival analysis of CDH4 and ADRM1 in TCGA colon cancer dataset. [file 12920_2020_776_MOESM2_ESM.zip › Additional File_2.docx]

**Figure S1**


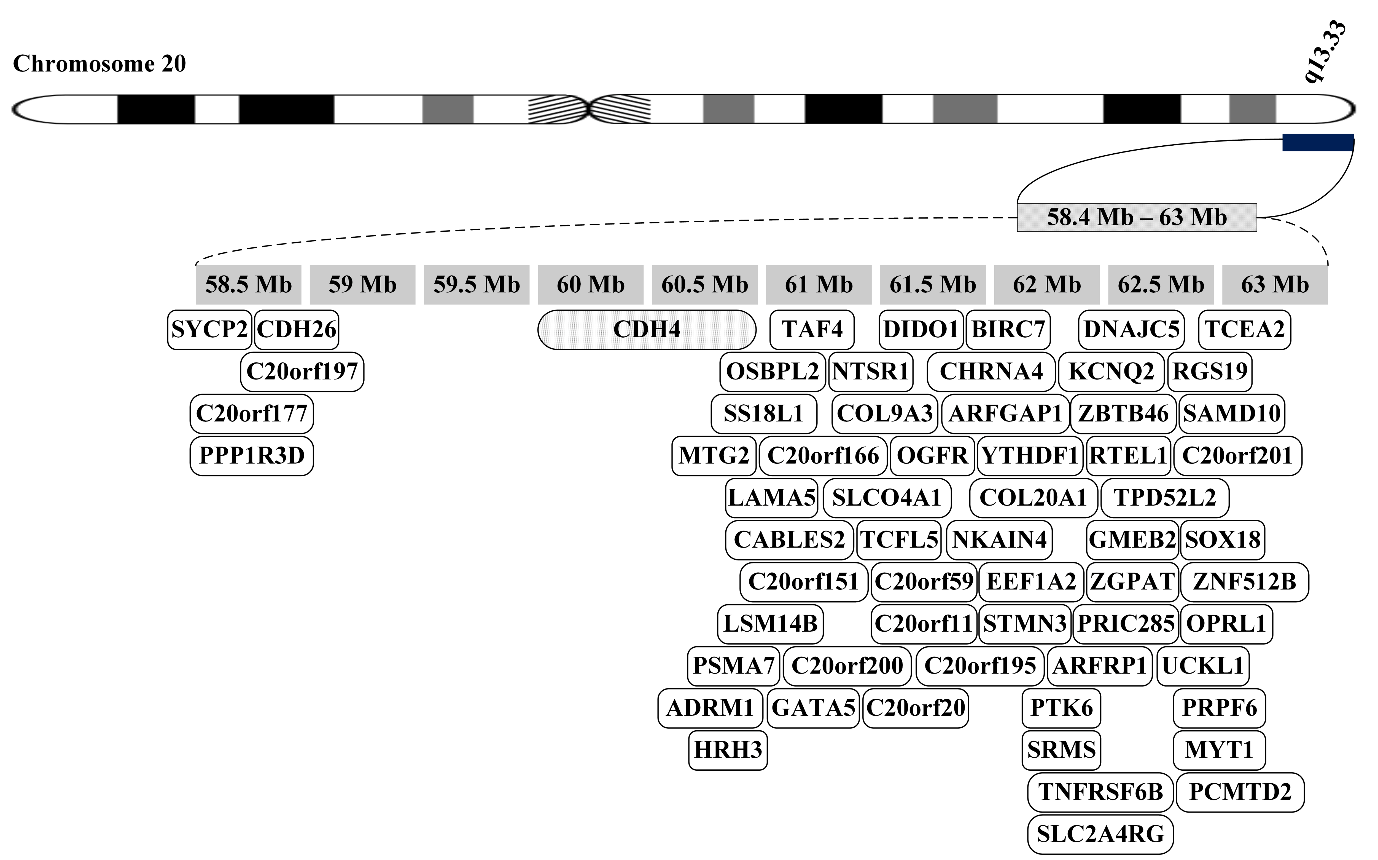


**Figure S1. Genome coordinates and annotated genes on chromosome 20q13.33 region.** There are 62 genes found in a 4.6 Mb region of chromosome 20q, and most of them are located within a 3 Mb interval (60 Mb – 63 Mb).

**Figure S2**

**A.**

**
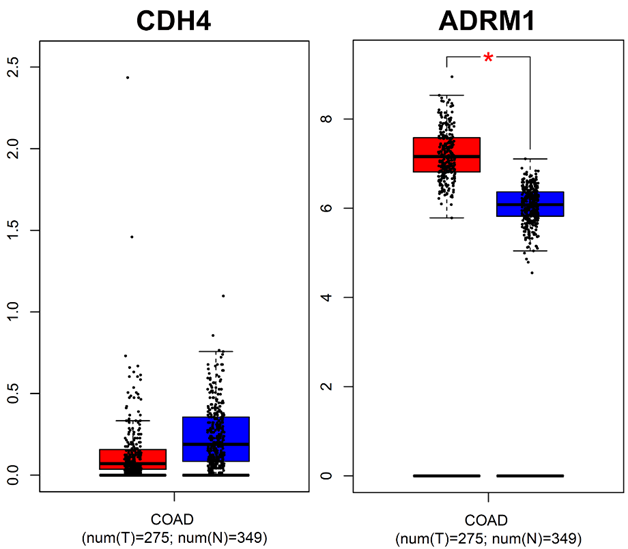
**

**B.**

**
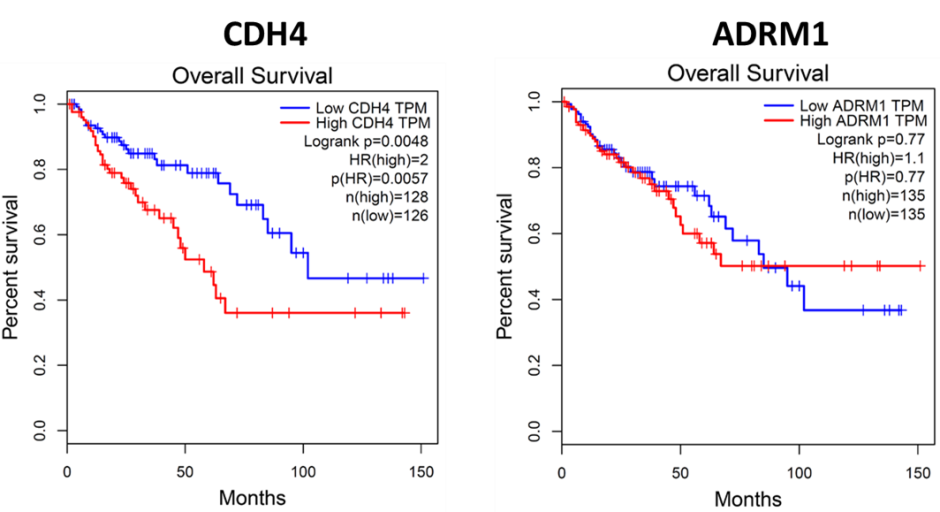
**

**Figure S2. Expression and survival analysis of CDH4 and ADRM1 in TCGA colon cancer dataset. A. Gene** expression was analyzed for CDH4 (left) and ADRM1 (right) using data from colon cancer (n=275) and normal colon (n=349) tissues in the TCGA database with GEPIA tool (http://gepia.cancer-pku.cn/index.html). **B.** Overall survival was analyzed from available data for CDH4 expression (high = 128; Low = 126) and ADRM1 expression (high = 135; Low = 135).
