# Supplementary figures and images for: Genomic amplification of chromosome 20q13.33 is the early biomarker for the development of sporadic colorectal carcinoma
Source: BMC Med Genomics. 2020 Oct 22;13(Suppl 10):149. doi: 10.1186/s12920-020-00776-z (PMC7579792; doi:10.1186/s12920-020-00776-z)

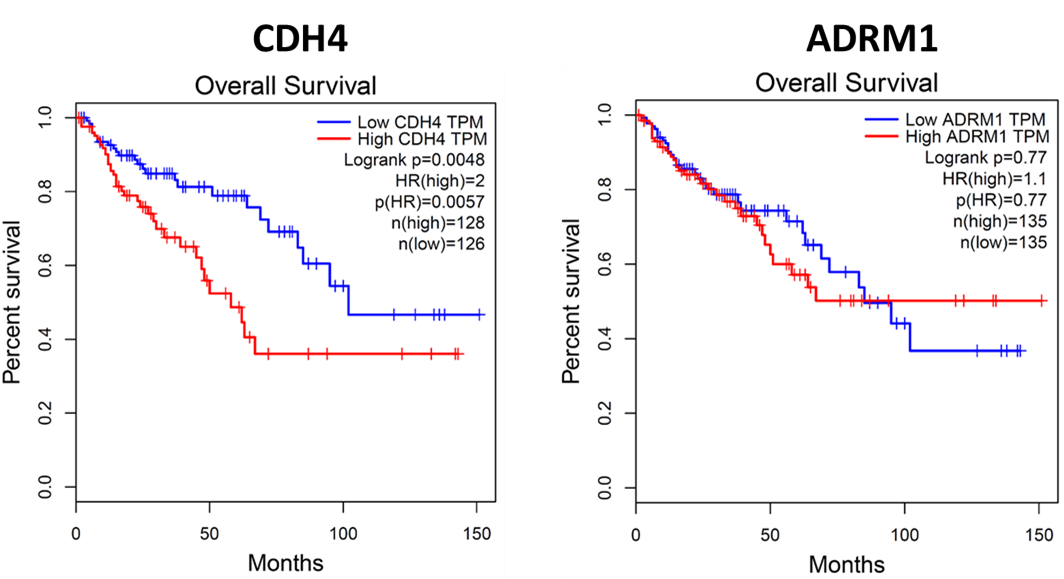

Supplement: Supplementary file 2 — Additional file 2: Figures S1. Genome coordinates and annotated genes on chromosome 20q13.33 region; and S2. Expression and survival analysis of CDH4 and ADRM1 in TCGA colon cancer dataset. [file 12920_2020_776_MOESM2_ESM.zip › Additional File 2_S2_B.tif]

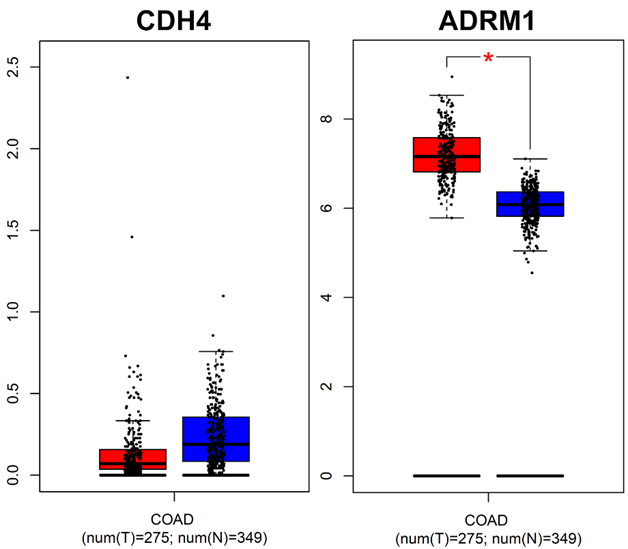

Supplement: Supplementary file 2 — Additional file 2: Figures S1. Genome coordinates and annotated genes on chromosome 20q13.33 region; and S2. Expression and survival analysis of CDH4 and ADRM1 in TCGA colon cancer dataset. [file 12920_2020_776_MOESM2_ESM.zip › Additional File 2_S2_A.tif]

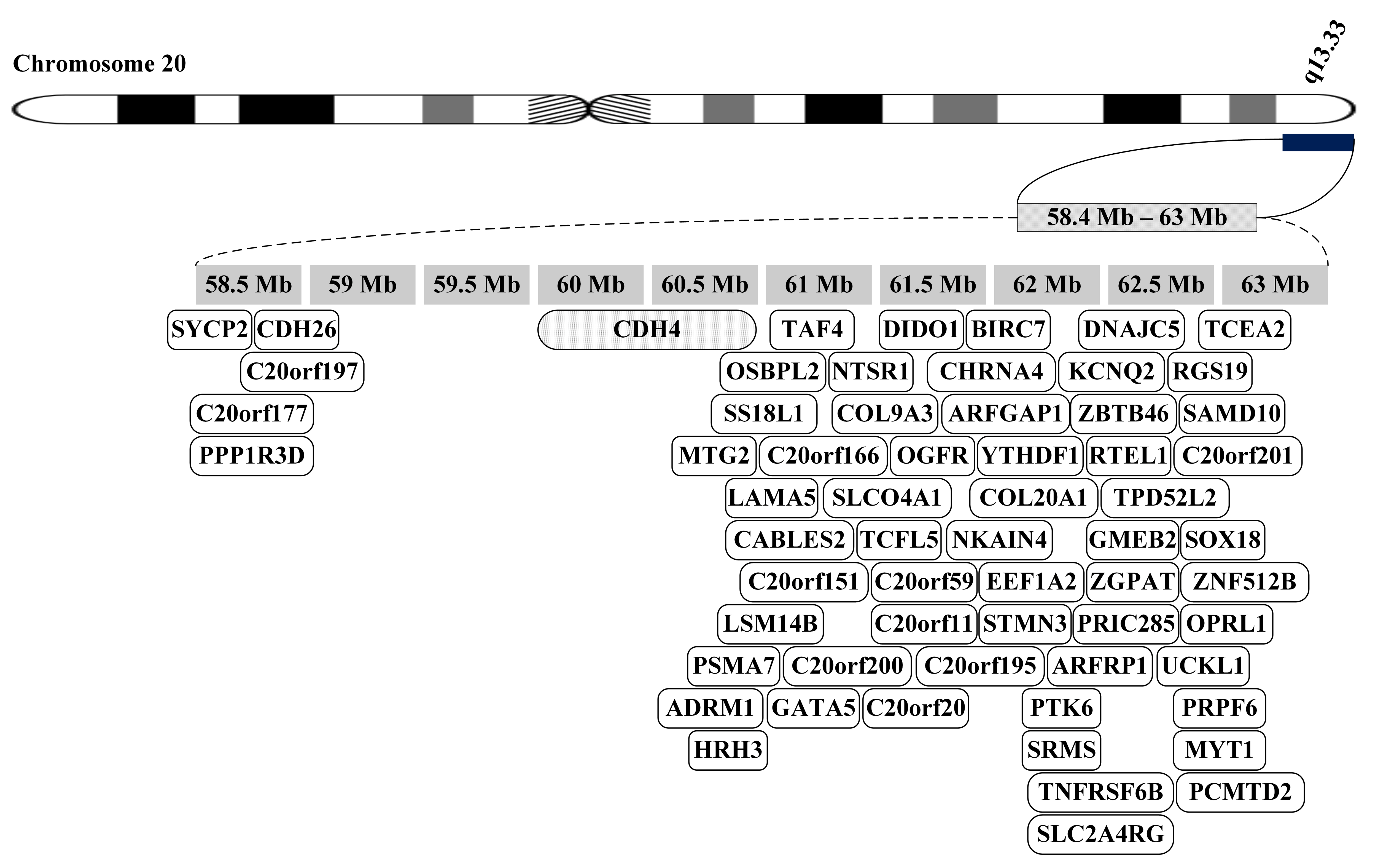

Supplement: Supplementary file 2 — Additional file 2: Figures S1. Genome coordinates and annotated genes on chromosome 20q13.33 region; and S2. Expression and survival analysis of CDH4 and ADRM1 in TCGA colon cancer dataset. [file 12920_2020_776_MOESM2_ESM.zip › Additional File 2_S1.tif]
