# Supplementary material for: Genomic amplification of chromosome 20q13.33 is the early biomarker for the development of sporadic colorectal carcinoma
Source: BMC Med Genomics. 2020 Oct 22;13(Suppl 10):149. doi: 10.1186/s12920-020-00776-z (PMC7579792; doi:10.1186/s12920-020-00776-z)
Supplement: Supplementary file 3 — Additional file 3: Table S2. Frequencies of copy number alterations in 4 target regions. [file 12920_2020_776_MOESM3_ESM.docx]

**Table S2.** Frequencies of copy number alterations in 4 target regions

| **Target region** | **Sample** | **Copy number alterations ^a^** | | |
| --- | --- | --- | --- | --- |
|  |  |  | **Number (%)** |  |
|  |  | **Gain** | **No change** | **Loss** |
| 20q13.33 (*CDH4*) | Non-tumor | 5 (1.3) | 335 (88.9) | 37 (9.8) |
|  | Tumor | 192 (50.9) | 159 (42.2) | 26 (6.9) |
|  | Population | 0 | 94 (100.0) | 0 |

**^a^** Total number of CRC samples = 377; Total number of the Han Chinese population = 94)
