# Supplementary material for: Genomic amplification of chromosome 20q13.33 is the early biomarker for the development of sporadic colorectal carcinoma
Source: BMC Med Genomics. 2020 Oct 22;13(Suppl 10):149. doi: 10.1186/s12920-020-00776-z (PMC7579792; doi:10.1186/s12920-020-00776-z)
Supplement: Supplementary file 4 — Additional file 4: Table S3. Detailed clinical information and 20q13.33 copy number estimation of CRC sample showing 20q13.33 copy number loss in their non-tumor part. [file 12920_2020_776_MOESM4_ESM.docx]

**Table S3.** Detailed clinical information and 20q13.33 copy number estimation of CRC sample showing 20q13.33 copy number loss in their non-tumor part

| Case number | ID | Gender | Age | AJCC stage | Location of tumor (a) | Differentiation of tumor | MS status | 20q13.33 copy number loss | | | |
| --- | --- | --- | --- | --- | --- | --- | --- | --- | --- | --- | --- |
|  |  |  |  |  |  |  |  | **Non-tumors** | | **Blood samples** | |
|  |  |  |  |  |  |  |  | **CN (b)** | **pCN (c)** | **CN (b)** | **pCN (c)** |
| 1 | 87 | Male | 74 | IIA | Right | Moderate | MSI-H | 1.34 | 1 | 2.13 | 2 |
| 2 | 218 | Female | 88 | IIIB | Right | Moderate | MSI-H | 1.34 | 1 | 2.14 | 2 |
| 3 | 291 | Female | 77 | IIIC | Right | Moderate | MSI-H | 1.03 | 1 | 2.36 | 2 |
| 4 | 305 | Female | 47 | IIIB | Right | Moderate | MSI-H | 1.16 | 1 | 2.23 | 2 |
| 5 | 175 | Male | 65 | I | Left | Moderate | MSI-L | 0.97 | 1 | 2.22 | 2 |
| 6 | 177 | Female | 87 | IIA | Right | Moderate | MSI-L | 0.97 | 1 | 2.22 | 2 |
| 7 | 185 | Female | 58 | IIIC | Right | Moderate | MSI-L | 1.19 | 1 | 2.15 | 2 |
| 8 | 211 | Female | 88 | IIA | Right | Moderate | MSI-L | 0.76 | 1 | 2.19 | 2 |
| 9 | 212 | Female | 89 | IIIC | Right | Moderate | MSI-L | 1.07 | 1 | 2.10 | 2 |
| 10 | 307 | Female | 76 | IIIB | Left | Moderate | MSI-L | 0.71 | 1 | 2.22 | 2 |
| 11 | 92 | Male | 75 | IIIB | Left | Moderate | MSS | 1.15 | 1 | 2.13 | 2 |
| 12 | 136 | Female | 54 | IIIC | Right | Moderate | MSS | 0.85 | 1 | 2.09 | 2 |
| 13 | 176 | Female | 68 | IIIB | Left | Moderate | MSS | 1.20 | 1 | 2.19 | 2 |
| 14 | 187 | Male | 76 | IIIB | Left | Moderate | MSS | 1.16 | 1 | 2.13 | 2 |
| 15 | 197 | Male | 55 | I | Right | Moderate | MSS | 1.37 | 1 | 2.42 | 2 |
| 16 | 214 | Male | 71 | I | Left | Well | MSS | 1.18 | 1 | 1.96 | 2 |
| 17 | 217 | Female | 65 | IIIB | Left | Moderate | MSS | 0.86 | 1 | 2.21 | 2 |
| 18 | 220 | Female | 46 | IIA | Left | Moderate | MSS | 0.89 | 1 | 2.06 | 2 |
| 19 | 233 | Male | 66 | IIA | Left | Well | MSS | 1.36 | 1 | 2.17 | 2 |
| 20 | 235 | Female | 56 | IIIB | Left | Moderate | MSS | 1.33 | 1 | 2.25 | 2 |
| 21 | 238 | Female | 52 | IIIB | Left | Well | MSS | 0.86 | 1 | 2.25 | 2 |
| 22 | 242 | Female | 73 | IIA | Right | Well | MSS | 0.76 | 1 | 2.45 | 2 |
| 23 | 269 | Female | 69 | IIIB | Left | Moderate | MSS | 0.74 | 1 | 2.24 | 2 |
| 24 | 287 | Female | 50 | IIIB | Left | Moderate | MSS | 0.73 | 1 | 2.30 | 2 |
| 25 | 297 | Male | 60 | I | Left | Moderate | MSS | 1.18 | 1 | 2.26 | 2 |
| 26 | 300 | Male | 75 | IIIB | Right | Moderate | MSS | 1.16 | 1 | 2.42 | 2 |

(a): all parts of right colon (such as appendix, cecum, ascending, hepatic flexure) and transverse colon were coded as right colon; the remains (included rectum) were coded as left colon

(b): Raw values of copy number were collected after quantitative real-time PCR

(c): Predicted values of copy number were calculated based on cutoff values
