# Supplementary material for: Genomic amplification of chromosome 20q13.33 is the early biomarker for the development of sporadic colorectal carcinoma
Source: BMC Med Genomics. 2020 Oct 22;13(Suppl 10):149. doi: 10.1186/s12920-020-00776-z (PMC7579792; doi:10.1186/s12920-020-00776-z)
Supplement: Supplementary file 5 — Additional file 5: Table S4. Primers used in this study. [file 12920_2020_776_MOESM5_ESM.docx]

**Table S4.** Primers used in this study

| **Primer name** | **Gene name** | **Sequences (5’ – 3’)** | **Size (bp)** |
| --- | --- | --- | --- |
| BAT-25_F | *c-kit* | /56-TAMN/TCGCCTCCAAGAATGTAAGT | 124 |
| BAT-25_R |  | TCTGCATTTTAACTATGGCTC |  |
| BAT-26_F | *hMSH2* | TGACTACTTTTGACTTCAGCC | 120 |
| BAT-26_R |  | /56-FAM/AACCATTCAACATTTTTAACCC |  |
| NR-21_F | *SLC7A8* | TAAATGTATGTCTCCCCTGG | 103 |
| NR-21_R |  | /5HEX/ATTCCTACTCCGCATTCACA |  |
| NR-22_F | *TPPB5* | GAGGCTTGTCAAGGACATAA | 142 |
| NR-22_R |  | /56-FAM/AATTCGGATGCCATCCAGTT |  |
| NR-24_F | *ZNF-2* | CCATTGCTGAATTTTACCTC | 132 |
| NR-24_R |  | /5HEX/ATTGTGCCATTGCATTCCAA |  |
| KRAS_F | *KRAS* | GGTACTGGTGGAGTATTTGATAGTG | 288 |
| KRAS_R |  | CATGAAAATGGTCAGAGAAACC |  |
| Codons 1260-1359  APC-A1F | *APC* | CAGACTTATTGTGTAGAAGA | 295 |
| APC-A2R |  | CTCCTGAAGAAAATTCAACA |  |
| Codons 1339-1516  APC-B1F | *APC* | AGGGTTCTAGTTTATCTTCA | 533 |
| APC-C2R |  | AAATGGCTCATCGAGGCTCA |  |
| Codons 1497-1596  APC-D1F | *APC* | ACTCCAGATGGATTTTCTTG | 300 |
| APC-D2R |  | GGCTGGCTTTTTTGCTTTAC |  |
